# Glycine confers neuroprotection through microRNA-301a/PTEN signaling

**DOI:** 10.1186/s13041-016-0241-3

**Published:** 2016-05-26

**Authors:** Juan Chen, Yang Zhuang, Zhi-Feng Zhang, Shu Wang, Ping Jin, Chunjiang He, Peng-Chao Hu, Ze-Fen Wang, Zhi-Qiang Li, Guang-Ming Xia, Gang Li, Yuan Wang, Qi Wan

**Affiliations:** Department of Physiology, School of Basic Medical Sciences, Medical Research Institute, Wuhan University School of Medicine, 185 Donghu Street, Wuhan, 430071 China; Department of Neurology, the Central Hospital of Wuhan, Wuhan, 430060 China; Department of Genetics, School of Basic Medical Sciences, Wuhan University School of Medicine, 185 Donghu Street, Wuhan, 430071 China; Department of Neurosurgery, Zhongnan Hospital, Wuhan University School of Medicine, 169 Donghu Street, Wuhan, 430071 China; Department of Neurology, the Central Hospital of Huanggang, Huanggang, 438000 China

**Keywords:** miR-301a, Glycine, PTEN, Neuroprotection

## Abstract

**Background:**

Glycine is known to protect against neuronal death. However, the underlying mechanism remains to be elucidated. The microRNA-301a is involved in both biological and pathological processes. But it is not known whether microRNA-301a has a neuroprotective property. In this study, we aimed to determine whether glycine-induced neuroprotection requires microRNA-301a-dependent signaling.

**Results:**

We provided the first evidence that glycine increased the expression of microRNA-301a in cultured rat cortical neurons and protected against cortical neuronal death through up-regulation of microRNA-301a after oxygen-glucose deprivation. MicroRNA-301a directly bound the predicted 3′UTR target sites of PTEN and reduced PTEN expression in cortical neurons. We revealed that PTEN down-regulation by microRNA-301a mediated glycine-induced neuroprotective effect following oxygen-glucose deprivation.

**Conclusions:**

Our results suggest that 1) microRNA-301a is neuroprotective in oxygen-glucose deprivation-induced neuronal injury; 2) glycine is an upstream regulator of microRNA-301a; 3) glycine confers neuroprotection through microRNA-301a/PTEN signal pathway.

## Background

Glycine is the simplest non-essential amino acid, which is a critical building block in many proteins. Glycine is essential for the synthesis of many biomolecules such as creatine, porphyrins and purine nucleotides. Glycine plays a fundamental role in cell metabolism [1, 2]. In the adult CNS, glycine is a major inhibitory neurotransmitter that binds to glycine receptor (a chloride-permeable ion channel) to inhibit postsynaptic neurons [3–7]. Interestingly, glycine is also a co-agonist of the excitatory NMDA receptor, a calcium-permeable ion channel in the CNS [8, 9]. Glycine has been shown to have neuroprotective effect in a variety of experimental models including ischemia–reperfusion injury, anoxia, hypoxia, ROS, chemically induced energy depletion [10–13]. In double-blinded, placebo-controlled clinical trial, glycine treatment shows significantly improved outcome and tended to decrease the 30 day mortality of stroke patients [14]. However, how glycine exerts its neuroprotective effect is largely unknown.

The tumor suppressor PTEN (phosphatase and tensin homolog deleted on chromosome 10) is a dual-specificity phosphatase [15, 16]. PTEN is involved in the regulation of several basic cellular functions, such as cell cycle progression, cell migration, cell spreading and cell growth [17]. We have previously shown that suppressing PTEN confers neuroprotection [18]. Down-regulation of PTEN is shown to protect against neuronal death through the enhancement of Akt activation, the inhibition of NR2B-containing NMDA receptors [18], the preservation of GABA_A_ receptors and the up-regulation of TDP-43 [19, 20]. We have also shown that NR2A-containing NMDA receptors and DJ-1 exert their neuroprotective effects by down-regulating PTEN [20, 21]. Consistent with our findings, recent studies have revealed that PTEN down-regulation is neuroprotective in cerebral ischemia, traumatic CNS injury and axon regeneration [22–36].

MicroRNAs (miRNAs) are a class of non-coding RNAs (∼22 nucleotides) that negatively regulate protein expression [37, 38]. MiRNAs regulate the expression of at least one third of the human genome and play a critical role in a variety of normal biological processes including cell differentiation, apoptosis, cell development and cell metabolism [37, 39, 40]. In animals, miRNAs regulate mRNA translation via imperfect pairing with nucleotide sequences within the 3′-untranslated region (3′UTR) of target genes [41].

MiRNA-301a (miR-301a) is the member of miR-130/301a family. miR-301a has been shown to involve in some biological and pathological processes, including cell development, cell differentiation, inflammation, apoptosis and cancer [42–45]. miR-301a is up-regulated in pancreatic cancer and to activate NF-kB by negative regulation of NF-kB-repressing factor gene [46]. miR-301a contributes to IL-6-induced insulin resistance by direct regulation of PTEN and downstream Akt signaling [47]. Recent studies demonstrate that miR-301a promotes cancer cell metastasis in breast, hepatocellular and gastric tumors through different target genes [43, 44, 48]. However, it is not clear whether miR-301a plays a role in neuronal survival.

In the present study, we demonstrate that glycine-induced neuroprotection depends on miR-301a and its downstream signaling. We show that the expression of miR-301a is elevated by glycine. miR-301a mediates glycine-induced neuroprotective effect in cultured cortical neurons subjected to oxygen-glucose deprivation (OGD). miR-301a binds the predicted 3′UTR target sites of PTEN and reduces PTEN expression. We provide evidence that glycine-induced neuroprotection is mediated through the up-regulation of miR-301a and subsequent suppression of PTEN expression.

## Results

### Glycine is neuroprotective in OGD-induced cortical neuronal injury

**Fig. 1:**
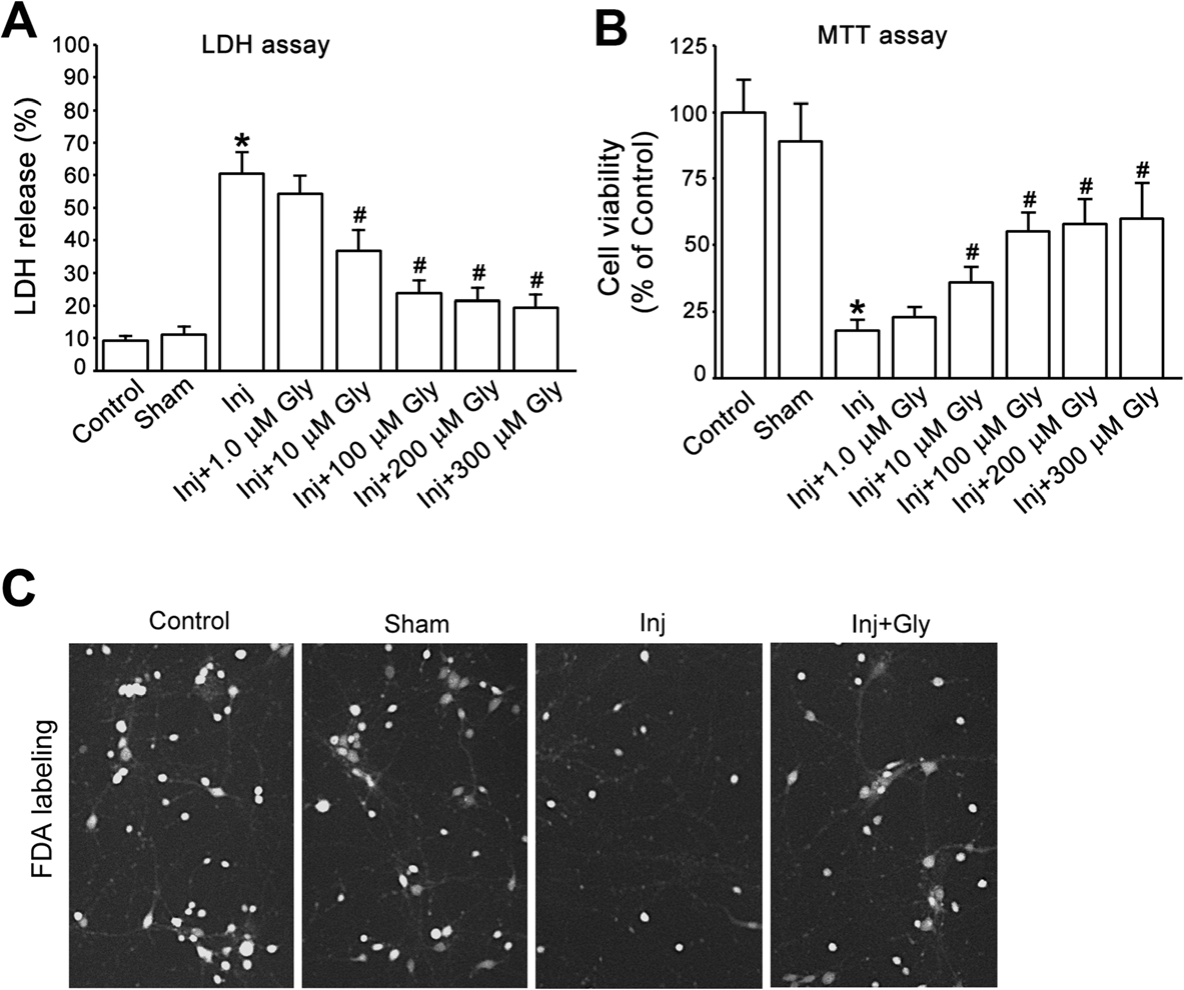
Glycine protects against OGD-induced cortical neuronal death. **a**, Glycine (10–300 μM) reduces OGD-induced increase of LDH release (*n =* 6, *P < 0.05 vs. Sham; ^#^ *P <* 0.05 vs. Inj, ANOVA test). **b**, MTT assay shows that glycine (10–300 μM) increases neuronal survival rate in OGD-induced cortical neuronal injury (*n =* 6, *P < 0.05 vs. Sham; ^#^ *P <* 0.05 vs. Inj, ANOVA test). **c**, Representative images of FDA labelling in cortical neuronal cultures showing that glycine (100 μM) reduces OGD induced-neuronal death. Inj, injury; Gly, glycine

Glycine has been shown to protect against OGD-induced neuronal injury. To verify the neuroprotective role of glycine in our experimental conditions, we examined the effect of glycine on cultured cortical neurons subjected to OGD. The cultured cortical neurons were injured by OGD for 1 h. At 1 h after OGD insult the cortical neurons were treated with glycine (100 μM). We treated the neurons with glycine at 1 h after OGD, instead of before OGD, in this study because of its clinical relevance. It is generally accepted that ischemic stroke treatment is required to be done within 4.5 h after ischemia/reperfusion onset. The treated neurons were collected for cell death and viability assays at 24 h after OGD insult. As shown in Fig. 1, LDH, MTT and FDA labeling assays showed that treatment of glycine reduced OGD-induced cortical neuronal death. These results support a neuroprotective role of glycine in OGD-induced neuronal injury.

### Glycine increases miR-301a expression in cortical neurons

**Fig. 2:**
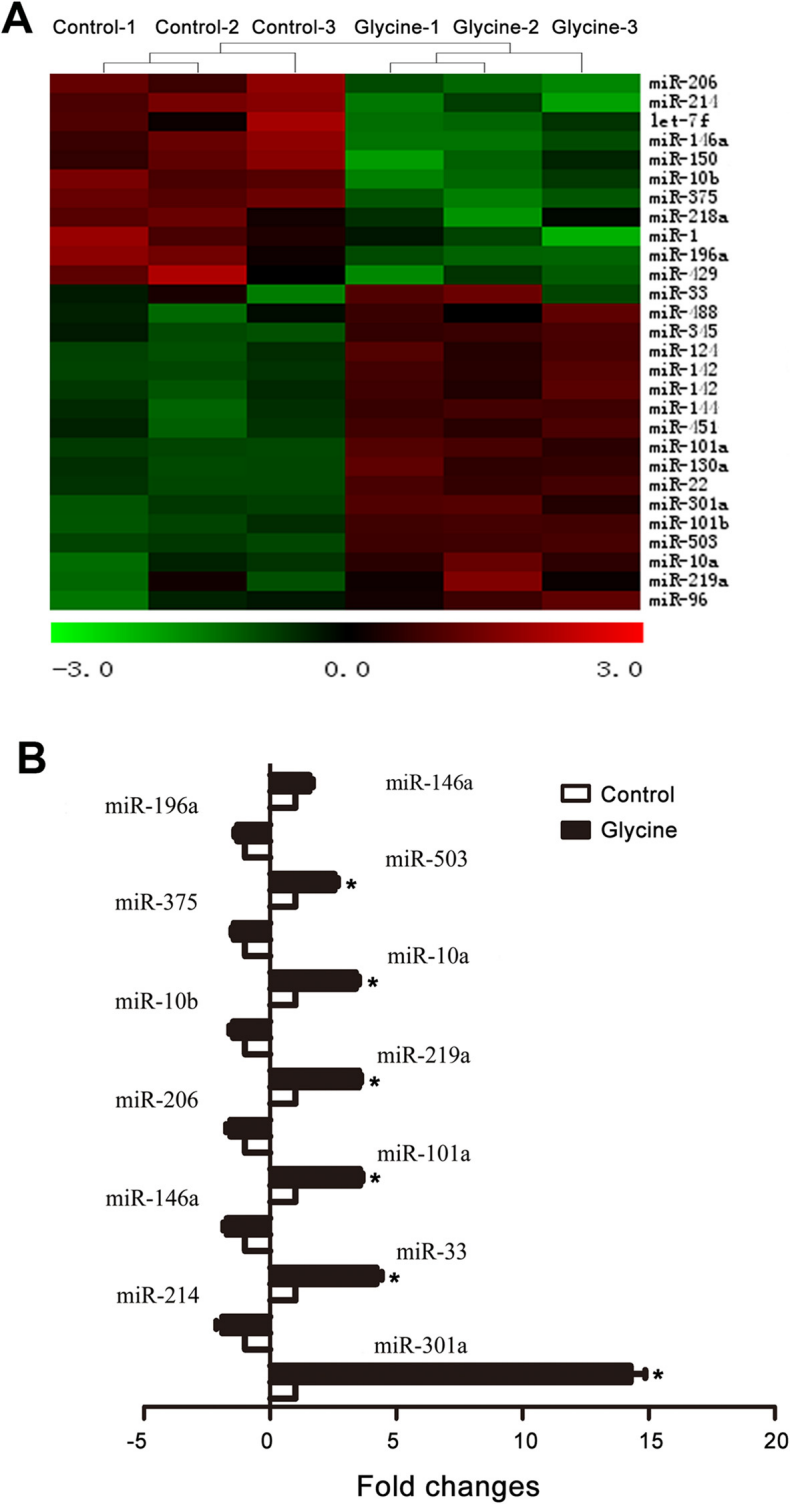
Glycine-induced increase of miR-301a expression in cortical neurons by microarray analysis. **a**, Heat map diagram showing differentially expressed miRNAs in glycine (100 μM)-treated cortical neurons. The cortical neurons isolated from 3 rats were used for the assay. **b**, The data of microarray assay show that the expression of miR-301a is remarkably increased in glycine (100 μM)-treated cortical neurons (*n =* 3, *P < 0.05 vs. Control, ANOVA test). The levels of miR-301a detected by microarray were normalized to a negative control of U6 snRNA

To determine whether glycine exerts its neuroprotective effect through regulating miRNA-dependent signaling, we performed microarray to analyze the miRNA expression profiles in cultured rat cortical neurons following glycine treatment. The cultured neurons were treated with or without glycine (100 μM) for 24 h and then collected for miRNA microarray assays. Total RNAs were isolated from glycine-treated cortical neurons and the miRNA microarray profiling of rat miRNA genes was performed (Fig. 2a). We found that glycine treatment resulted in differential changes in the expression of miRNAs in the cortical neurons (Fig. 2a), among which the miR-301a was markedly increased (Fig. 2b).

**Fig. 3:**
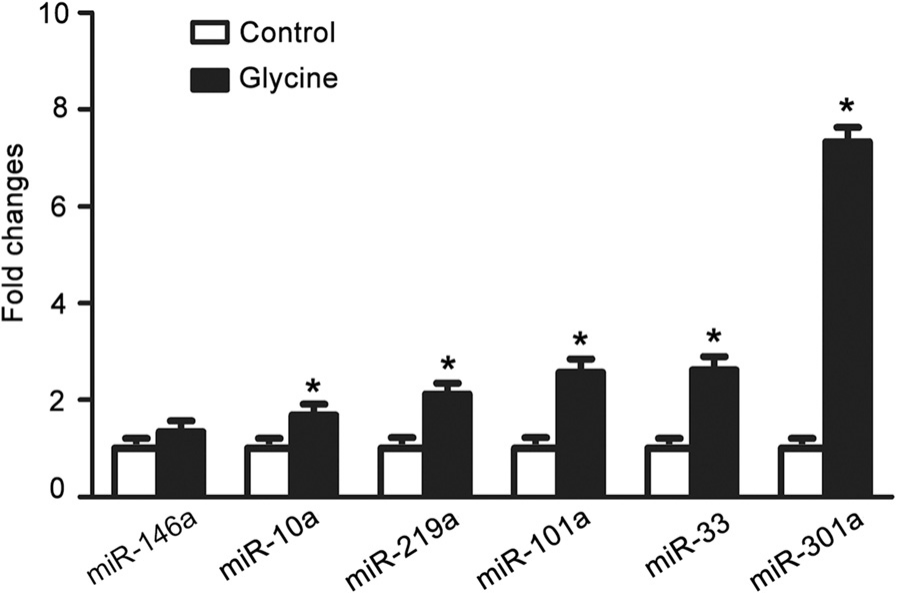
Verification of glycine-induced increase of miR-301a by qRT-PCR. qRT-PCR reveals that treatment of glycine (100 μM) for 24 h increased the expression of miR-301a in cultured cortical neurons (*n =* 6, *P < 0.05 vs. Control, ANOVA test). The levels of miRNAs were calculated using U6 snRNA as an internal control

To provide further evidence to verify glycine-induced increase of miR-301a, qRT-PCR was used to measure the miR-301a expression after the rat cortical neurons were treated with glycine (100 μM) for 24 h. As expected, the qRT-PCR results confirmed that glycine enhanced the expression of miR-301a in the cortical neurons (Fig. 3).

### miR-301a is neuroprotective in OGD-induced neuronal injury

**Fig. 4:**
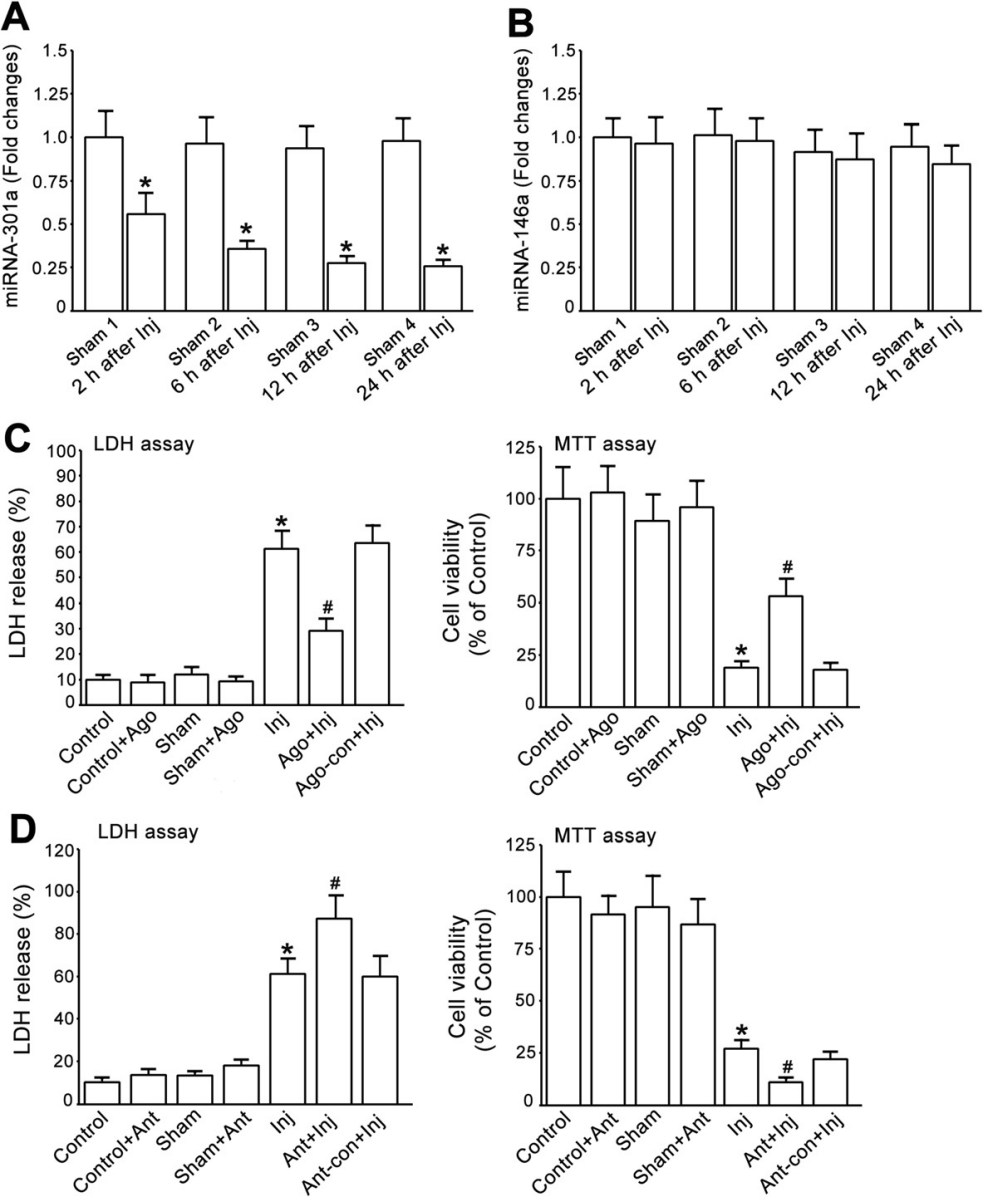
miR-301a protects against OGD-induced cortical neuronal death. **a**, The expression of miR-301a in cultured cortical neurons at different time following OGD insult are measured by qRT-PCR. miR-301a is decreased at 2, 6, 12 & 24 h after OGD insult (*n =* 3, *P < 0.05 vs. Sham, ANOVA test). The data are normalized to that in Sham 1. **b**, The expression of miR-146a in cultured cortical neurons at different time following OGD insult are measured by qRT-PCR. miR-146a is not altered at 2, 6, 12 & 24 h after OGD insult (*n =* 3, ANOVA test). The data are normalized to that in Sham 1. **c**, Treatment of miR-301a agomir reduces OGD-induced cortical neuronal death. Left, LDH assay; Right, MTT assay (*n =* 6, *P < 0.05 vs. Sham, ^#^ *P <* 0.05 vs. Inj, ANOVA test). **d**, Treatment of miR-301a antagomir increases OGD-induced neuronal death. Left, LDH assay; Right, MTT assay (*n =* 6, *P < 0.05 vs. Sham, ^#^ *P <* 0.05 vs. Inj, ANOVA test). Inj, injury; Gly, glycine; Ant, antagomir; Ant-con, antagomir control; Ago, agomir; Ago-con, agomir control

Given the neuroprotective effect of glycine, the observed up-regulation of miR-301a by glycine led us to reason that miR-301a may play a neuroprotective role in neuronal injury. We first set up to measure the response of endogenous miR-301a to neuronal injury. We examined the expression of miR-301a in cultured cortical neurons at different time after OGD insult. The qRT-PCR data revealed that the expression of miR-301a, but not miR-146a, was decreased at 2, 6, 12 and 24 h after OGD-induced injury in cultured cortical neurons (Fig. 4a, b).

To determine whether miR-301a is neuroprotective, the cortical neurons were treated with miR-301a agomir or agomir control. At 24 h after the treatment, the neurons were subjected to OGD for 1 h. At 24 h after OGD insult, we found that treatment of miR-301a agomir, but not the agomir control, protected against OGD-induced neuronal death after OGD insult (Fig. 4c). In the same experimental protocols, treatment of miR-301a antagomir but not antagomir control increased OGD-induced neuronal death (Fig. 4d). These results provides the first evidence that miR-301a is neuroprotective in neuronal injury.

### miR-301a is required for glycine-induced neuroprotection

**Fig. 5:**
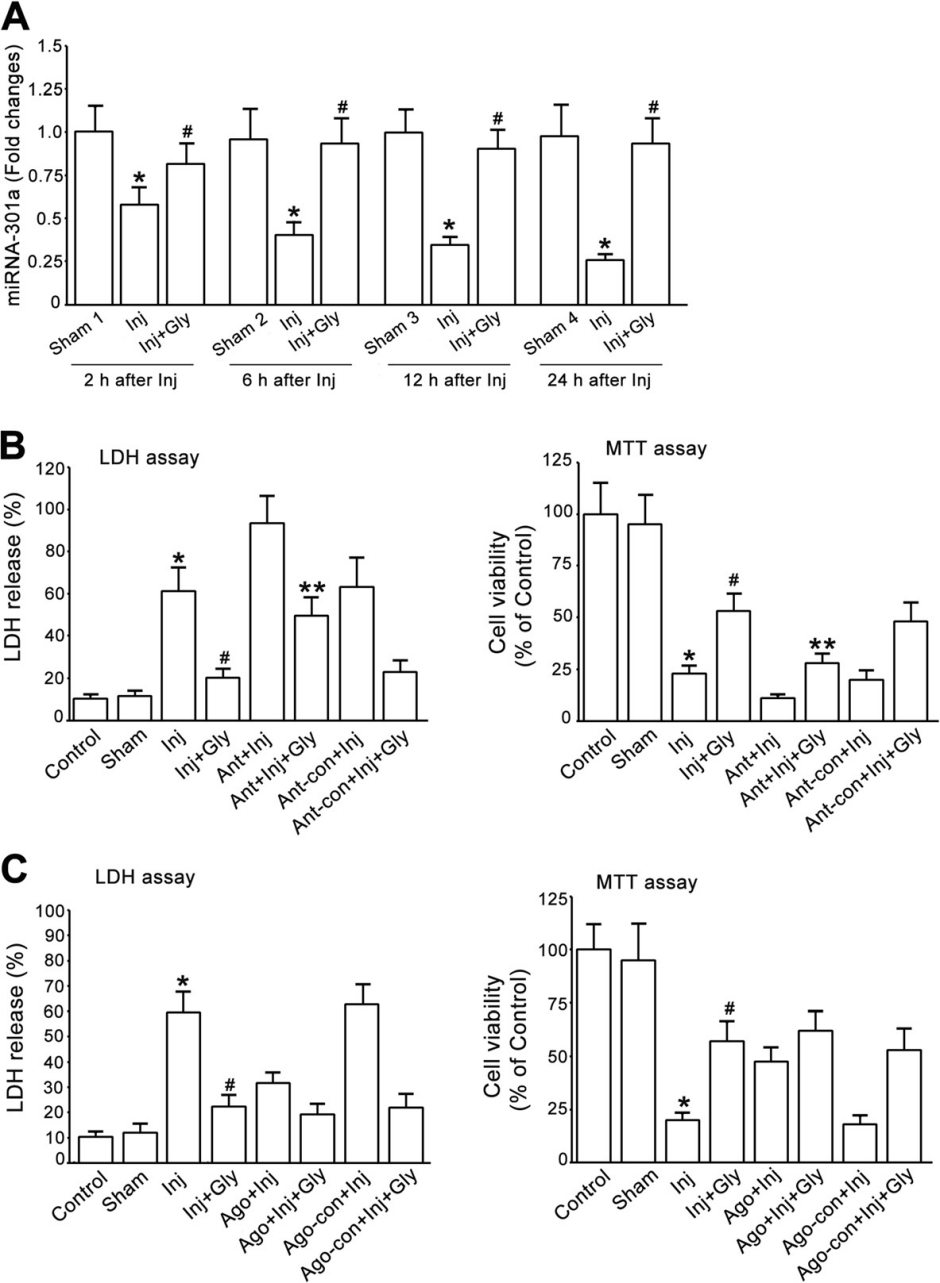
miR-301a mediates glycine-induced neuroprotective effect. **a**, Glycine treatment increases miR-301a expression after OGD (*n =* 3, *P < 0.05 vs. Sham, ^#^ *P <* 0.05 vs. Inj, ANOVA test). **b,** Treatment of miR-301a antagomir, but not antagomir control, attenuates glycine-induced neuroprotection against LDH release in cultured cortical neurons subjected to OGD insult. Left, LDH assay; Right, MTT assay (*n =* 6, *P < 0.05 vs. Sham, ^#^ *P <* 0.05 vs. Inj, **P < 0.05 vs. Inj + Gly, ANOVA test). **c**, Treatment of miR-301a agomir in cultured cortical neurons does not enhance glycine-induced neuroprotective effect after OGD insult. Left, LDH assay; Right, MTT assay (*n =* 6, *P < 0.05 vs. Sham, ^#^ *P <* 0.05 vs. Inj + Gly, ANOVA test). Inj, injury; Gly, glycine; Ant, antagomir; Ant-con, antagomir control; Ago, agomir; Ago-con, agomir control

To determine whether glycine-induced neuroprotection is mediated through miR-301a, we first measured the effects of glycine on the expression of miR-301a in cultured cortical neurons at different time following OGD insult. The neurons were subjected to OGD for 1 h and treated with glycine (100 μM) at 1 h after OGD. The qRT-PCR data showed that glycine prevented OGD-induced decrease of miR-301a at 2, 6, 12 and 24 h after injury in cultured cortical neurons (Fig. 5a). To provide evidence whether glycine-induced increase of miR-301a conferred neuroprotection, the cortical neurons were treated with miR-301a antagomir or antagomir control. At 24 h after treatment, the treated neurons were subjected to OGD for 1 h. At 1 h after OGD injury the cortical neurons were treated with glycine (100 μM) and collected for cell death and viability assays at 24 h after OGD insult. We showed that suppression of miR-301a by miR-301a antagomir attenuated glycine-induced neuroprotective effect (Fig. 5b). To provide further evidence, we treated the neurons with miR-301a agomir or agomir control. At 24 h after the treatment, the neurons were subjected to OGD for 1 h and then treated with glycine (100 μM). At 24 h after OGD insult, we showed that the neuroprotective efficiency induced by glycine in agomir-treated neurons was not significantly higher than that in agomir control-treated neurons (Fig. 5c), suggesting that miR-301a is in the same pathway with glycine to exert neuroprotective effect. Collectively, these data indicate that glycine confers neuroprotection through enhancing miR-301a expression.

### PTEN is a target gene of miR-301a

**Fig. 6:**
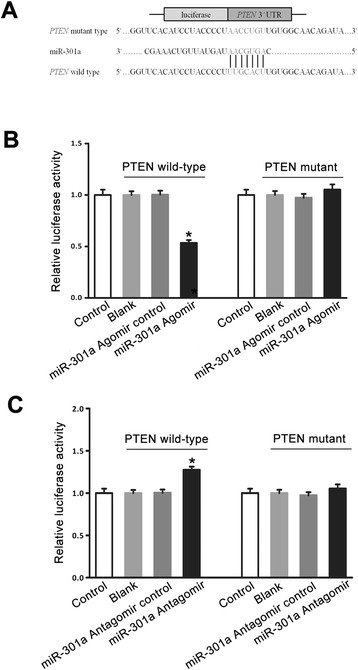
miR-301a directly binds the predicted 3′UTR target sites of PTEN. **a**, The predicted miR-301a target sequence in the 3′UTR target sites of the PTEN gene. A mutation was generated in the seed region of PTEN 3′UTR as indicated by the underline. **b**, Luciferase reporter assays in PC12 cells transfected with pMIR-REPORT Luciferase vector carrying PTEN 3′UTR (PTEN wild-type) versus pMIR-mut-PTEN 3′UTR (PTEN mutant) in the absence or presence of miR-301a agomir. The luciferase activity was reduced in the PC12 cells co-transfected with a luciferase reporter containing the PTEN 3′UTR and miR-301a agomir (*n =* 6, *P < 0.05 vs. miR-301a agomir control, ANOVA test). **C**, Luciferase reporter assays in PC12 cells transfected with pMIR-REPORT Luciferase vector carrying PTEN 3′UTR (PTEN wild-type) versus pMIR-mut-PTEN 3′UTR (PTEN mutant) in the absence or presence of the miR-301a antagomir. The luciferase activity was increased in the PC12 cells co-transfected with a luciferase reporter containing the pMIR-PTEN 3′UTR and miR-301a antagomir (*n =* 6, *P < 0.05 vs. miR-301a antagomir control, ANOVA test). Control: untransfected PC12 cells; Blank: PC12 cells transfected with pMIR-REPORT Luciferase vector carrying PTEN wild-type or PTEN mutant without miR-301a agomir or antagomir

To explore how miR-301a exerts its effect in mediating glycine-induced neuroprotection, we analyzed the target genes of miR-301a by miRanda (www.microrna.org), TargetScan (www.targetscan.org) and miRDB (mirdb.org) and predicted PTEN as a target of miR-301a. As shown in Fig. 6a, miR-301a was predicted to target the 2206–2212 nts of PTEN 3′UTR. To determine whether miR-301a directly binds the predicted 3′UTR sites of PTEN, we made a reporter construct harbouring the 589 bp fragment of PTEN 3′UTR flanking the entire putative target sequence. We performed luciferase assay and showed that ectopic expression of miR-301a agomir resulted in a significant reduction of luciferase activity in the PC12 cells (Fig. 6b). The mutation of the seed sequence of miR-301a within the 3′UTR of PTEN abrogated the inhibition of luciferase activity by exogenous miR-301a agomir in PC12 cells (Fig. 6b). In contrast, the luciferase activity was increased in the PC12 cells co-transfected with a luciferase reporter containing the PTEN-3′UTR (pMIR-PTEN 3′UTR) and miR-301a antagomir (Fig. 6c). These results suggest that miR-301a directly binds the predicted 3′UTR target sites of PTEN.

### PTEN is regulated by miR-301a in cortical neurons

**Fig. 7:**
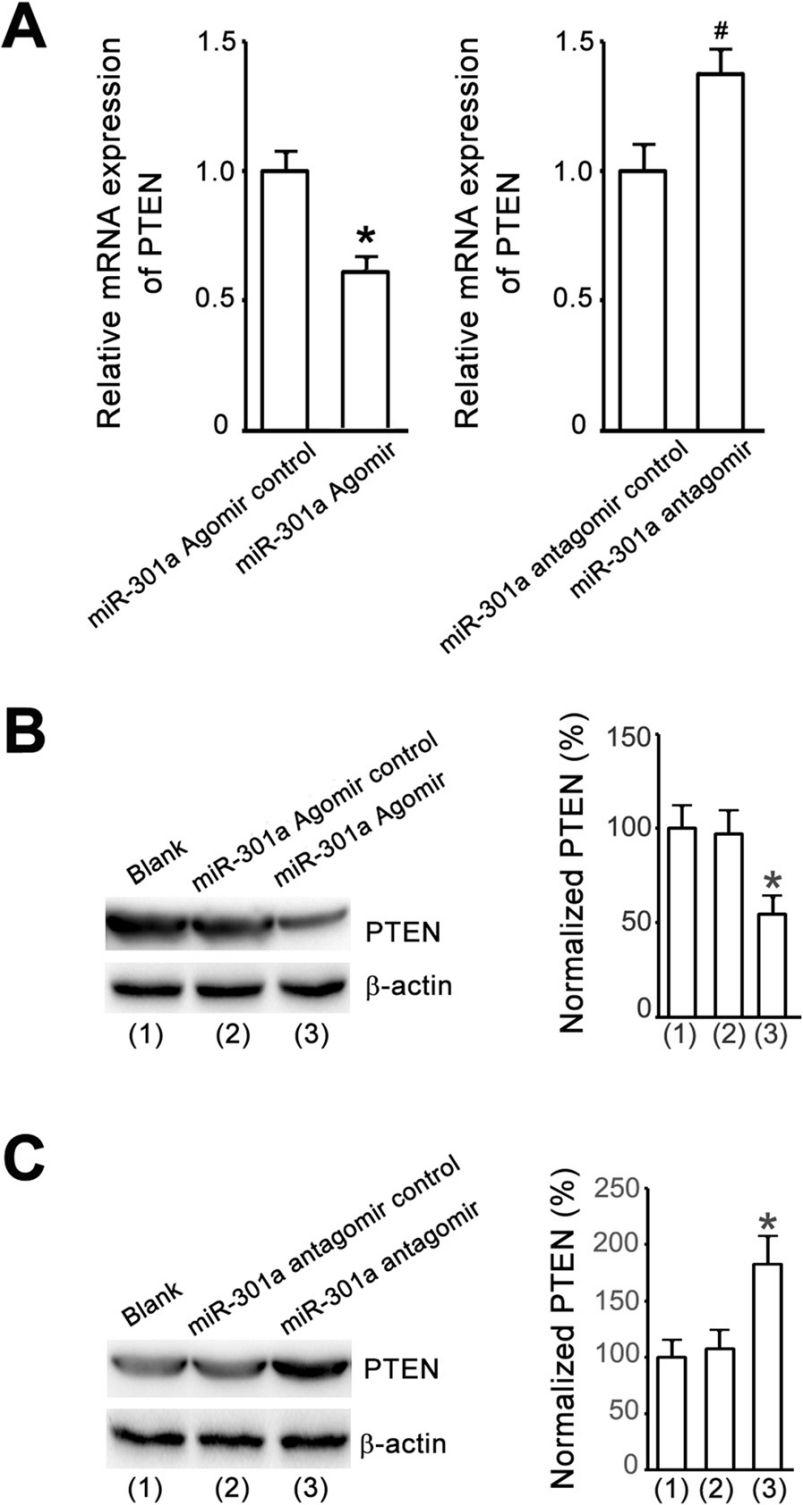
miR-301a regulates PTEN expression. **a**, qRT-PCR shows that the levels of PTEN mRNAs are down-regulated and up-regulated in the cortical neurons treated with miR-301a agomir and antagomir, respectively (*n =* 6, *P < 0.05 vs. agomir control, ^#^ *P <* 0.05 vs. antagomir control, Student *t* test). **b**, Western blot assay shows that the levels of PTEN proteins in the cortical neurons treated with miR-301a agomir are reduced (*n =* 6, *P < 0.05 vs. agomir control, ANOVA test). **c,** Western blot assay shows that the levels of PTEN proteins in the cortical neurons treated with miR-301a antagomir are increased (*n =* 6, *P < 0.05 vs. antagomir control, ANOVA test)

To determine the functional consequence of miR-301a binding to PTEN, we tested the effects of miR-301a agomir or antagomir on the mRNA and protein expression of PTEN in cortical neurons. We demonstrated that the level of PTEN mRNA was down-regulated in the cultured cortical neurons treated with miR-301a agomir for 24 h and up-regulated in the cultured cortical neurons treated with miR-301a antagomir for 24 h (Fig.7a). Western blot analysis further showed that 24 h treatment of miR-301 agomir decreased the protein expression of PTEN but 24 h treatment of miR-301 antagomir increased the level of PTEN proteins in cultured cortical neurons (Fig. 7b-c). These data indicate that miR-301 negatively regulates the mRNA and protein expression of PTEN in cortical neurons.

### miR-301a suppresses PTEN to mediate glycine-induced neuroprotection

**Fig. 8:**
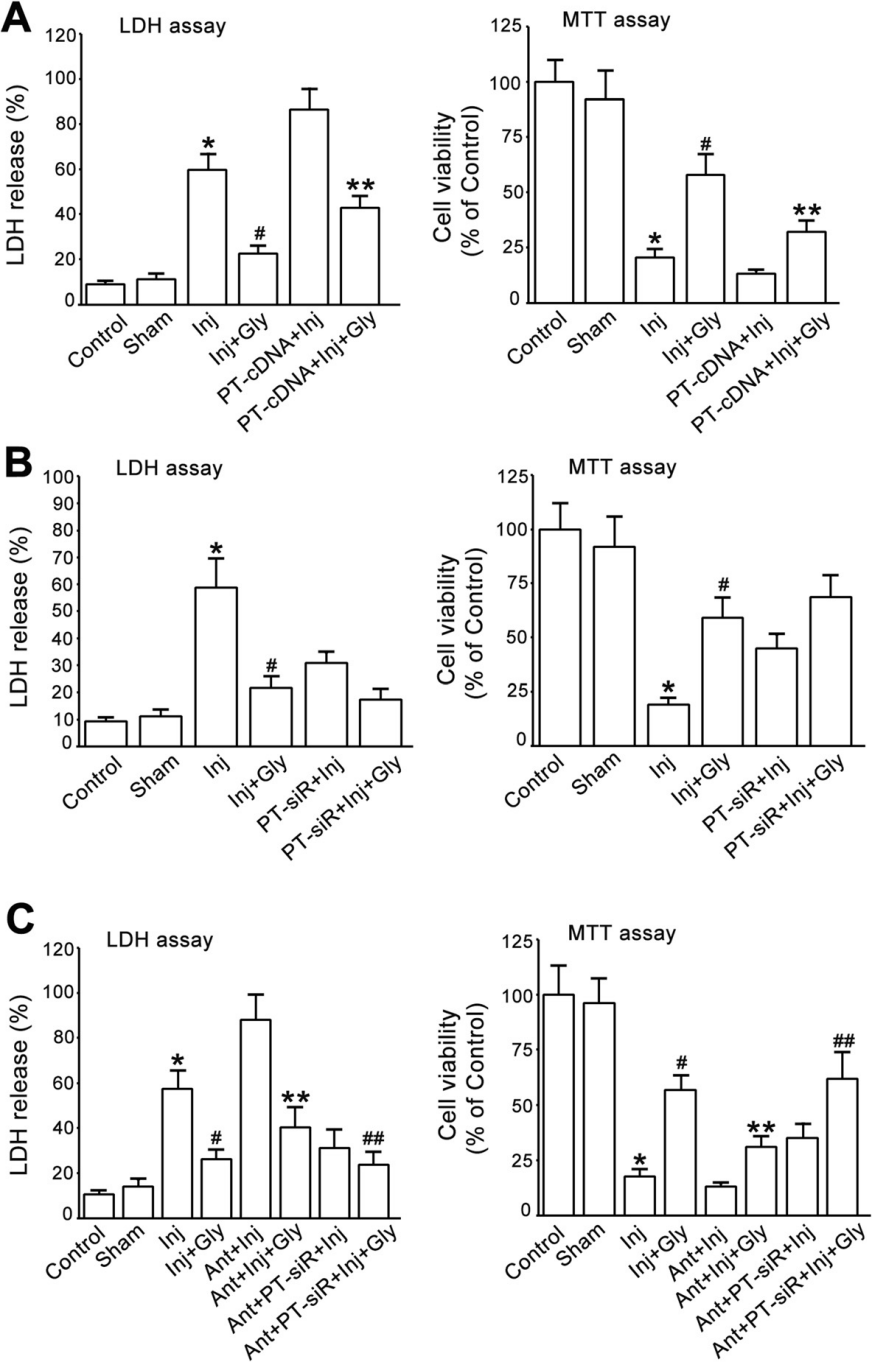
miR-301a mediates glycine-induced neuroprotection through suppression of PTEN. **a**, Transfection of PTEN cDNA reduces glycine-induced neuroprotection in cultured cortical neurons subjected to OGD insult. Left, LDH assay; Right, MTT assay (*n =* 6, *P < 0.05 vs. Sham, ^#^ *P <* 0.05 vs. Inj, **P < 0.05 vs. Inj + Gly, ANOVA test). **b**, Suppression of PTEN by PTEN siRNA in cultured cortical neurons does not increase glycine-induced neuroprotective effect in OGD insult (*n =* 6, *P < 0.05 vs. Sham, ^#^ *P <* 0.05 vs. Inj, ANOVA test). Left, LDH assay; Right, MTT assay (*n =* 6, *P < 0.05 vs. Sham, ^#^ *P <* 0.05 vs. Inj, ANOVA test). **c**, PTEN siRNA treatment prevents miR-301a antagomir from blocking glycine-induced neuroprotection. Left, LDH assay; Right, MTT assay (*n =* 6, *P < 0.05 vs. Sham, ^#^ *P <* 0.05 vs. Inj, **P < 0.05 vs. Inj + Gly, ^##^ *P <* 0.05 vs. Ant + Inj + Gly, ANOVA test). Inj, injury; Gly, glycine; Ant, antagomir; Ant-con, antagomir control; Ago, agomir; Ago-con, agomir control; PT-cDNA, PTEN cDNA; PT-siR, PTEN siRNA

We next tested whether miR-301a mediates glycine-induced neuroprotection through PTEN down-regulation. The cultured cortical neurons were transduced with lentiviral vectors encoding PTEN cDNAs. At 48 h after transduction, the neurons were subjected to OGD for 1 h. At 1 h after OGD injury the cortical neurons were treated with glycine (100 μM) and collected for cell death and viability assays at 24 h after OGD insult. We showed that overexpression of PTEN reduced glycine-induced neuroprotection (Fig. 8a).

The cortical neurons were also transduced with lentiviral PTEN siRNAs. At 48 h after transduction, the neurons were subjected to OGD for 1 h. At 1 h after OGD insult the neurons were treated with glycine (100 μM) and collected for cell death and viability assays at 24 h following OGD insult. We found that pre-suppression of PTEN by the siRNA did not significantly enhance glycine-induced neuroprotective effect (Fig. 8b).

The cortical neurons were then treated with lentiviral PTEN siRNA or miR-301a antagomir. The neurons were first treated with lentiviral PTEN siRNA. At 24 h after the transduction, the neurons were treated with miR-301a antagomir or antagomir control. At 24 h after the treatment of antagomir or antagomir control, the neurons were subjected to OGD for 1 h. At 1 h after OGD insult the neurons were treated with glycine (100 μM) and collected for cell death and viability assays at 24 h after OGD insult. We demonstrated that PTEN down-regulation by PTEN siRNA prevented miR-301a antagomir from blocking glycine-induced neuroprotection (Fig. 8c). Taken together, we conclude that miR-301a targets PTEN to mediate glycine-induced neuroprotection.

## Discussion

Glycine is shown to confer protection against neuronal injuries in both in vitro and in vivo experimental conditions [10–13, 49, 50]. Importantly, clinical trial has shown that glycine treatment improves outcome of ischemic stroke patients [14]. However, how glycine exerts its neuroprotective effect remains to be elucidated. The present study provides new evidence that the neuroprotective effect of glycine is mediated by the enhancement of miR-301a expression and subsequent suppression of PTEN expression in cortical neurons. These findings reveal a molecular mechanism by which miRNA-dependent signaling mediates glycine-induced neuroprotection. It is not yet clear how miR301a is regulated by glycine. The expression level of biologically active mature miRNAs is the result of a fine mechanism of biogenesis, carried out by different enzymatic complexes that exert their function at transcriptional and post-transcriptional levels [51]. It is possible that glycine may regulate the processing of miRNA biogenesis. In the cytoplasm, Dicer is an RNase III that digests the pre-miRNA into a 20–25 nucleotides mature duplex miRNA [51]. For example, TAp63, a p53 family member, has been reported to coordinately regulate Dicer and miR-130b to suppress metastasis [52]. TAp63 binds to and transactivates the Dicer promoter, resulting in a direct transcriptional regulation of Dicer by TAp63 [52]. We would reason that glycine may regulate miR301a through a transcriptional mechanism by which Dicer is modulated. Further studies will be performed to test this hypothesis.

As glycine is a co-agonist of NMDA receptors, regulation of miR301a by glycine may be mediated by NMDA receptor-dependent signaling. Previous study has provided evidence that the activation of GluN2A-containing NMDA receptors, but not GluN2B-containing NMDA receptors, contributes to glycine-induced neuroprotective effect in ischemic injury [10, 50]. These studies show that glycine induces its neuroprotection through enhancement of Akt activation and CREB phosphorylation. Thus, it is necessary to investigate next whether GluN2A-containing NMDA receptor-dependent Akt/forkhead/FOXO and/or CREB signaling mediate glycine-induced modulation of miR-301a.

Recent studies indicate that miR-301a plays a critical role in tumorigenesis [43, 44, 46, 48]. It has been reported that miR-301a is up-regulated in hepatocellular carcinoma and modulates NF-kB expression by negatively regulating Gax [53]. miR-301a is also up-regulated in gastric tumor cells and involved in the clinical progression and prognosis of gastric cancer [45]. miR-301a promotes migration and invasion by targeting TGFBR2 in human colorectal cancer [48]. The up-regulation of miR-301a in breast cancer and Ewing’s sarcoma cells promotes tumor metastasis and tumor cell proliferation by targeting the tumor suppressor PTEN [44, 54]. Through negative regulation of SMAD4, miR-301a promotes pancreatic cancer progression [55]. In addition to its effect on cancer, miR-301a is shown to play an important role in regulating the expression of Kv4.2 in diabetes [56] and controlling autoimmune demyelination by regulating the T-helper 17 immune response [57]. Our study for the first time demonstrates that miR-301a exerts a neuroprotective effect in OGD-induced neuronal injury, implicating a therapeutic potential of miR-301a signaling in neurological diseases. Interestingly, we reveal that glycine acts as an upstream regulator to enhance miR-301a expression, which confer neuroprotection in cortical neurons.

We and others have previously revealed that down-regulation of PTEN is neuroprotective in cerebral ischemia, traumatic CNS injury and axon regeneration [16, 18, 20, 22–36]. We have shown for the first time that depressing PTEN protects against ischemic neuronal death through the enhancement of Akt activation, the inhibition of NR2B-containing NMDA receptors, and the preservation of GABA_A_ receptors [18, 19]. We also show that PTEN suppression prevents excitotoxicity-induced neuronal death via the up-regulation of nuclear TDP-43 [20]. To understand how PTEN is regulated in the neuronal injury process, we have revealed that NR2A-containing NMDA receptors and DJ-1 act as the negative regulators of PTEN to exert their neuroprotective effects [21]. In the present study, we provide new evidence that PTEN is negatively regulated by miR-301a and that PTEN suppression by miR-301a up-regulation mediates glycine-induced neuroprotective effect. Thus, we identify a molecular mechanism by which the glycine/miR-301a signal pathway negatively regulates PTEN in conferring neuroprotection.

## Methods

### Neuronal culture and OGD insult

The cortical neuronal cultures were prepared from Sprague–Dawley rats at gestation day 17 as described [58, 59]. Briefly, dissociated neurons were suspended in plating medium (Neurobasal medium, 2 % B-27 supplement, 0.5 % FBS, 0.5 μM L-glutamine, and 25 μM glutamic acid) and plated on poly-D-lysine coated Petri dishes. After 1 day in culture, half of the plating medium was removed and replaced with maintenance medium (Neurobasal medium, 2 % B-27 supplement, and 0.5 μM L-glutamine). Thereafter, maintenance medium was changed in the same manner every 3 days. The cultured neurons were used for experiments at 12 days after plating.

To initiate the OGD challenge, cells were transferred to deoxygenated glucose-free extracellular solution (ECS) (in mM: 116 NaCl, 5.4 KCl, 0.8 MgSO4, 1.0 NaH2PO4, 1.8 CaCl2, and 26 NaHCO3), introduced into a specialized chamber (Plas-Labs, Lansing, MI), and maintained at 37 °C in 85 % N2/10 % H2/5 % CO2 for 1 h. Neurons were removed from the chamber, transferred to maintenance medium, and returned to the incubator. For “sham” treatment, cultures were transferred to the ECS (in mM: 116 NaCl, 5.4 KCl, 0.8 MgSO4, 1.0 NaH2PO4, 1.8 CaCl2, 26 NaHCO3, and 33 glucose), introduced into the chamber, and maintained at 37 °C for 1 h in 95 % O2/5 % CO2. After the sham treatment, the neurons were transferred to maintenance medium and returned to the original incubator.

### LDH Assay

The lactate dehydrogenase (LDH) is a cytoplasmic enzyme retained by viable cells with intact plasma membranes and released from cells with damaged membranes. The LDH release was measured using CytoTox 96 Cytotoxicity kit based on the manufacturer’s instructions (Promega, USA). The levels of maximal LDH release were measured by treating the cultures with 10× lysis solution (provided by the manufacturer) to yield complete lysis of the cells. Absorbance data were obtained using a 96-well plate reader (Molecular Devices, USA) at 490 nm. According to the manufacturer’s instructions, the LDH release (%) was calculated by calculating the ratio of experimental LDH release to maximal LDH release.

### MTT Assay

The viability of the cells in the neuronal cultures was assessed by their ability to uptake thiazolyl blue tetrazolium bromide (MTT). The cells were incubated with MTT for 1 h, then lysed with dimethyl sulfoxide (DMSO) and left at room temperature in the dark overnight. The lysates were then read on a plate reader (PowerWave X, Bio-Tek) at the absorbance wavelength of 540 nm.

### Labeling of cortical neurons by fluorescein diacetate

Fluorescein diacetate (FDA) was performed to detect neuronal viability. Briefly, cultures were rinsed with extracellular solution and incubated with FDA (5 μM) for 30 min. The cultures were washed with extracellular solution and then viewed on an Olympus fluorescent microscope (IX51, Olympus).

### miRNA microarray assay

Cultured rat cortical neurons treated with or without glycine (100 μM) for 24 h were collected for miRNA microarray analysis. Total RNAs were isolated using TRIzol reagent (Invitrogen, USA) and a miRNeasy Mini Kit (QIAGEN, Germany). RNA samples were applied to the miScript miRNA PCR Arrays (QIAGEN, Germany). Expression profiling was done with dissociation curves using ABI QuantStudio 6 K Flex (ABI, USA). 500 ng RNA template, miScript Reverse Transcriptase Mix (2 μl), 5× miScript HiSpec Buffer (4 μl), 10× miScript Nucleics Mix (2 μl) added into RNase-free water to the volume of 20 μl. Reverse transcription reactions were incubated at 37 °C (60 min) followed by at 95 °C (5 min). The qPCR reactions were consisted of cDNA template (0.8 μl), 2× QuantiTect SYBR Green PCR Master Mix (12.5 μl), 10× miScript Universal Primer (2.5 μl), added into RNase-free water to the volume of 25 μl. Reactions were incubated at 95 °C (15 min), followed by 40 cycles at 94 °C (15 s), 55 °C (30 s) and 70 °C (30 s), followed by melting curve ananlysis. Fold changes in miRNA expression were calculated using the comparative Ct method and normalized to U6 small nuclear RNA (snRNA) as endogenous control. After normalization, differentially expressed miRNAs were identified through Fold Change filtering.

### Real-time qRT-PCR

For the quantification of miRNAs or mRNA, total RNAs were isolated using TRIzol reagent (Invitrogen, USA), the reverse transcribed using Maxima H Minus First Strand cDNA Synthesis Kit (Thermo, USA) and the qPCR reaction using SYBR Green qPCR Master mixture (ABI, USA). The reverse transcription was performed on Bio-Rad MJ Mini Instrument (Bio-Rad, USA) and qPCR was performed on QuantStudio 6 K Flex Instrument (ABI, USA).

For the quantification of miRNAs, 2 μg RNA template and 500 nM RT Primer (2 μl) added into RNase-free water to the volume of 11 μl, were incubated at 70 °C (10 min) followed by on ice (2 min), then 5× RT Buffer (5 μl), 2.5 mM dNTP Mix (2 μl), 100U RT enzyme (1 μl) added into RNase-free water to the volume of 25 μl. Reverse transcription reactions were incubated at 42 °C (60 min) followed by at 70 °C (10 min). The miRNA qPCR reactions were consisted of SYBR Green qPCR Master mixture (10 μl), cDNA template (2 μl), 5 μM Bulge-Loop miRNA Forward Primer (0.8 μl) and 5 μM Bulge-Loop miRNA Reverse Primer (0.8 μl) added into RNase-free water to the volume of 20 μl. Reactions were incubated at 95 °C (20 s), followed by 40 cycles at 95 °C (10 s), 60 °C (20 s) and 70 °C (10 s), followed by melting curve analysis.

For the quantification of mRNA, 2 μg RNA template, 25 pM oligo (dT) Primer (0. 25 μl), 10 mM dNTP Mix (1 μl), 5× RT Buffer (4 μl), Maxima H Minus Enzyme Mix (1 μl) were added into RNase-free water to the volume of 20 μl. Reverse transcription reactions were incubated at 25 °C (10 min) followed by at 50 °C (15 min) and at 85 °C (5 min). The qPCR reaction mixtures included the SYBR Green qPCR Master mixture (10 μl), cDNA template (2 μl) and 10 μM Forward Primer (0.6 μl) and 10 μM Reverse Primer (0.6 μl) added into RNase-free water to the volume of 20 μl. Reactions were incubated at 95 °C (3 min), followed by 40 cycles at 95 °C (10 s), 60 °C (30 s), followed by melting curve analysis. All reactions were performed in triplicate for each sample tested. The median Ct (Cycle threshold) value was used for analysis. Relative quantities were calculated using the 2^-△△Ct^ method with U6 snRNA/β-actin. Three independent experiments were performed.

### Constructs and treatment

The miR-301a agomir, agomir control, antagomir, antagomir control were purchased from RioBio (China). The cultures were washed with ECS for 10 min, and then treated with serum-free medium supplemented with 1.0 μM agomir, agomir control, antagomir or antagomir control for 2 h at 5 % CO2, 95 % humidity and 37 °C. After incubation, the cultures were further cultured in maintenance medium for 24 h [60].

The lentiviral PTEN cDNA particles and lentiviral PTEN siRNA particles were purchased from GeneChem (China). The treatment of particles was performed in cultured cortical neurons based on the manufacturer’s instructions.

### Luciferase assay

A 589 bp fragment of the PTEN mRNA-3′UTR was amplified from cDNA by PCR and cloned into pMIR-REPORT Luciferase vector (Invitrogen, USA) to make pMIR-PTEN-3′UTR reporter construct. The primes were 5′-GGACTAGT GGATTTACACATTTATATTTGAACA-3′(F) and 5′-CCCAAGCTT TACCTACAGAACGCAGTTCAGGCTA-3′(R). Site-directed mutagenesis was performed to disrupt the sequence within PTEN mRNA-3′UTR which was complementary to the miR-301a (MIMAT0000552) seed region, and the primers were purchased from RioBio (China). The mutated vector was named pMIR-mut-PTEN 3′UTR. The reporter constructs were confirmed by DNA sequencing.

A total of 5 × 10^4^ PC12 cells were seeded in 24-well plates for 24 h and then co-transfected with 100 ng pMIR-PTEN-3′UTR constructs using Lipofectamine 2000 (Invitrogen, USA) with 1.0 μM miR-301a agomir or agomir control, or with the miR-301a antagomir or antagomir control. Renilla luciferase pRL-TK reporter (Promega, USA) was co-transfected to monitor the transfection efficiency. At 24 h post-transfection, the luciferase activities were measured by the dual-luciferase reporter assay system using the Glomax2020 luminometer (Promega, USA). Data were normalized by Renilla luciferase. Each experiment was performed at least three times in triplicate wells.

### Western blotting

Western blotting assay was performed as described previously [18, 58]. For the detection of phospho-Akt, the samples prepared in the same day were used. The polyvinylidene difluoride membrane (Millipore, USA) was incubated with primary antibody against PTEN and β-actin (Cell Signaling, USA). Primary antibodies were labeled with horseradish peroxidase-conjugated secondary antibody, and protein bands were imaged using SuperSignal West Femto Maximum Sensitivity Substrate (Pierce, USA). The EC3 Imaging System (UVP, USA) was used to obtained blot images directly from the polyvinylidene difluoride membrane. The quantification of Western blot data was performed using ImageJ software.

### Statistical analysis

Student’s *t* test or ANOVA test was used where appropriate to examine the statistical significance of the differences between groups of data. Newman–Keuls tests were used for post-hoc comparisons when appropriate. All results are presented as mean ± SE. Significance was placed at *P <* 0.05.

## Conclusions

Our results indicate that microRNA-301a is neuroprotective in oxygen-glucose deprivation-induced neuronal injury. We provide evidence that glycine is an upstream regulator of microRNA-301a. We conclude that glycine confers neuroprotection through microRNA-301a/PTEN signal pathway.

## Abbreviations

miR-301a, microRNA-301a; OGD, oxygen-glucose deprivation; PTEN, phosphatase and tensin homolog deleted on chromosome 10; 3′UTR, 3′-untranslated region; LDH, lactate dehydrogenase; MTT, thiazolyl blue tetrazolium bromide; FDA, fluorescein diacetate
